# The Relationship Between the Cortico‐Diaphragmatic Conduction Pathway and Cardiopulmonary Function in Healthy Individuals

**DOI:** 10.1111/crj.70198

**Published:** 2026-05-24

**Authors:** Long‐ping Wang, Jing Chen, Cheng Wu, Shan‐tong Yao, Jin‐ze Tan, Shuang Guo, Qian Ding, Guang‐qing Xu

**Affiliations:** ^1^ Department of Rehabilitation Medicine Guangdong Provincial People's Hospital, Guangdong Academy of Medical Sciences, Southern Medical University Guangzhou Guangdong China; ^2^ School of Rehabilitation Medicine Shandong Second Medical University Weifang China; ^3^ Guangdong Cardiovascular Institute Guangdong Provincial People's Hospital, Guangdong Academy of Medical Sciences Guangzhou Guangdong China

**Keywords:** cardiopulmonary function, corticospinal diaphragm pathway, TMS

## Abstract

**Aims:**

To investigate whether there is a correlation between the parameters of cardiopulmonary function tests and corticospinal diaphragm pathway parameters in healthy individuals.

**Methods:**

Seventeen healthy adults participated in all tests. Transcranial magnetic stimulation (TMS) was employed to assess corticospinal diaphragm excitability, measuring the diaphragmatic resting motor threshold (DRMT), cortical motor evoked potential amplitude (CMEPA), and cortical motor evoked potential latency (CMEPL). Functional capacity was evaluated using the 6‐min walk test (6MWT), pulmonary ventilation function was assessed via pulmonary function tests (PFT), and cardiopulmonary endurance was determined by cardiopulmonary exercise test. (CPET). The relationships between TMS‐derived parameters and cardiopulmonary function indices were examined using correlation analysis and stepwise regression analysis.

**Results:**

Regression analyses identified two key independent predictors. First, forced expiratory volume in 1 s (FEV1) was an independent predictor of DRMT. DRMT also correlated with other measures of pulmonary function and exercise capacity. Second, maximum heart rate (HR_max_) was an independent predictor of CMEPL. CMEPL also showed positive correlations with other cardiopulmonary parameters.

**Conclusion:**

This study demonstrates that in healthy adults, better pulmonary function (reflected by higher FEV1) is associated with a higher DRMT and greater cardiovascular reserve (reflected by higher HR_max_) is associated with longer CMEPL. These novel associations suggest a link between routine cardiopulmonary metrics and the central neural control of breathing, warranting further investigation.

## Graphical abstract

Relationship between central control and cardiopulmonary function. Forced expiratory volume in the first second and the maximum heart rate is closely linked to corticospinal diaphragm pathway. Better cardiopulmonary function may experience inhibition of excitability in the diaphragmatic motor cortex.

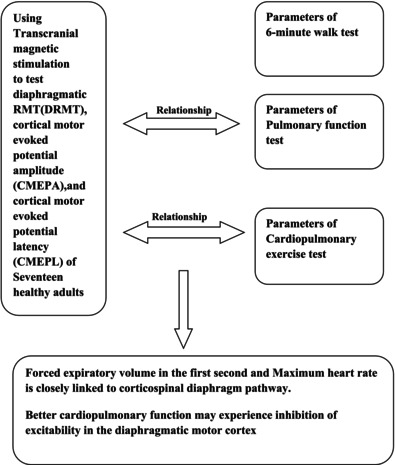

## 1 Introduction

The diaphragm serves as the primary respiratory muscle and plays a crucial role in maintaining normal breathing and cardiopulmonary function. Its activity is regulated by the voluntary respiratory control system in the cerebral cortex and the involuntary respiratory control system in the lower brainstem, including the medulla and pons [1]. This highly efficient operational mode of the diaphragm not only enhances pulmonary ventilation efficiency, but, more importantly, its rhythmic movement extensively modulates cardiopulmonary function through neurohumoral mechanisms. During its contraction, intrathoracic pressure decreases, facilitating lung expansion during inspiration. Relaxation of the diaphragm reduces thoracic volume, leading to passive exhalation. Diaphragmatic breathing promotes efficient oxygen exchange, which can slow the heart rate and may contribute to the reduction or stabilization of blood pressure [2].

Transcranial magnetic stimulation (TMS) is a safe and noninvasive technique used to assess and modulate motor function in the human brain. It has been widely applied across various neurological domains, including language, cognition, psychology, swallowing, and other motor functions [3]. When applied to the diaphragmatic motor cortex, TMS can elicit motor evoked potentials (MEPs) in the diaphragm, demonstrating its utility in evaluating the corticospinal excitability of the diaphragm, which has been confirmed in both human and animal studies [4, 5, 6]. In respiratory diseases, central respiratory drive is closely associated with dyspnea [7]. Studies indicate that the central cortical diaphragmatic motor system is affected during acute exacerbations of chronic obstructive pulmonary disease (AECOPD) [8], and this impairment correlates with pulmonary function. Therefore, the corticospinal diaphragm pathway may be a critical factor influencing diaphragm function. Research by Khedr et al. [9] on stroke patients revealed significant correlations between physiological parameters of the cortical‐diaphragmatic pathway and the degree of motor impairment, infarct location, diaphragmatic movement amplitude, and the extent of respiratory dysfunction. Furthermore, Miscio et al. used TMS to investigate the characteristics of the cortical‐diaphragmatic pathway in 14 patients diagnosed with amyotrophic lateral sclerosis (ALS). Although routine pulmonary function tests and gas analysis in these patients showed no abnormalities, eight exhibited reduced MEP values, four demonstrated delayed spinal MEPs (Sp‐MEPs), and one showed absent cortical MEPs (Cx‐MEPs) [10]. These findings suggest that assessment of the corticospinal diaphragm pathway may possess greater sensitivity than conventional cardiopulmonary function tests in detecting subclinical neurological involvement.

Although the aforementioned studies have revealed the potential of TMS in assessing alterations in the cortical‐diaphragm pathway in patients with conditions such as COPD, stroke, and ALS, most of these investigations have primarily focused on characterizing pathophysiological changes under specific disease states. Currently, systematic studies examining the associations between physiological parameters of this pathway (e.g., threshold, amplitude, and latency) and objective cardiopulmonary function test metrics remain relatively limited. This is particularly true in healthy adults, where the fundamental relationship remains unclear. Therefore, whether central assessment of the diaphragm can be developed into an effective tool for evaluating cardiopulmonary function remains an open question.

Therefore, this study aims to elucidate the characteristics of the corticospinal diaphragm pathway in healthy adults, with a specific focus on analyzing the correlations between TMS‐derived physiological parameters and cardiopulmonary function test indicators. The findings are expected to address existing knowledge gaps in this field and provide a foundation for understanding the neurophysiological mechanisms underlying related diseases, as well as developing novel assessment methodologies.

## 2 Materials and Methods

### 2.1 Subjects

Seventeen healthy adults (mean age: 21 years, range: 19–33) were recruited as participants, all of whom were right‐handed. The exclusion criteria comprised conditions that might affect central nervous system conduction or contraindications for TMS stimulation, including (i) history or current diagnosis of central nervous system disorders, (ii) implants or metallic materials in the head or neck region, (iii) cardiac pacemakers, and (iv) pregnancy or any cardiopulmonary diseases. The study protocol was approved by the Ethics Committee of Guangdong Provincial People's Hospital. All participants provided written informed consent.

### 2.2 Experimental Setup

#### 2.2.1 TMS

TMS was performed using a NS5000 Magnetic Stimulator (YIRUIDE Medical Company, Wuhan, China). TMS was applied over the diaphragmatic cortical representation area using a circular coil (70 mm diameter) that was positioned tangentially 45° from the midline to induce a posterior anterior current in the hemisphere. All stimuli were delivered using single‐pulse, biphasic waveform pulses.

#### 2.2.2 6‐Min Walk Test (6MWT)

The 6‐min walk test (6MWT) was performed in accordance with the American Thoracic Society guidelines. Participants were instructed to walk as fast as possible for 6 min along a 30‐m corridor configured as a round‐trip course around marker cones. During the test, heart rate, oxygen saturation, and walking distance were monitored and recorded using a Sensecho5A wearable device (Beijing SensEcho Technology Co. Ltd., Beijing, China) [11].

#### 2.2.3 Pulmonary Function Test (PFT)

This test was conducted in accordance with the latest pulmonary function test guidelines that were published by the European Respiratory Society (ERS) and the American Thoracic Society (ATS) [12]. Forced vital capacity (FVC), forced expiratory volume in the first second (FEV1), and peak expiratory flow (PEF) were measured using the AS‐600 pulmonary function instrument (Model AS‐600, Minato Corporation, Tokyo, Japan).

#### 2.2.4 Cardiopulmonary Exercise Test (CPET)

This test was conducted using a CORTEX cardiopulmonary exercise testing system (Metalyzer, Custo‐Med, Leipzig, Germany). The test protocol followed a ramp exercise paradigm with minute‐by‐minute workload increments. The rate of increase, the change in workload (ΔW), was calculated using the formula: ΔW = [predicted peak oxygen uptake (VO_2_, mL/min) − predicted unloaded VO_2_ (mL/min)], where predicted peak VO_2_ (mL/min) = [height (cm) − age] × 20 for males or ×14 for females, and predicted unloaded VO_2_ (mL/min) = 150 + [6 × body weight (kg)]. Pedaling cadence was maintained between 55 and 65 rpm. The test comprised a 3‐min resting phase, a 3‐min unloaded warm‐up phase, a ramp loading phase (6–10 min), and a 3‐min unloaded recovery phase. The gas analysis system was calibrated prior to each test using standard gases for both volume and gas concentration. Test termination criteria were based on established standards [13], including (1) attainment of 85%–100% of the age‐predicted maximum heart rate (calculated as 220 − age); (2) significant arrhythmias, such as ventricular tachycardia or sustained supraventricular tachycardia; (3) marked blood pressure abnormalities, defined as systolic pressure > 250 mmHg or < 90 mmHg, or diastolic pressure > 115 mmHg; (4) development of respiratory or cardiovascular symptoms, including chest pain, dyspnea, vertigo, or syncope; (5) participant's request to stop due to extreme fatigue; (6) significant desaturation, with peripheral oxygen saturation falling below 88%; and (7) respiratory exchange ratio (RER) reaching or exceeding 1.15. These criteria ensured maximal participant effort. Of the 17 participants, 10 met the predetermined heart rate criterion for test termination, whereas 7 discontinued due to reaching maximal fatigue levels. After the test, we recorded parameters such as maximum work rate (WRmax), maximum oxygen uptake (VO_2max_), oxygen consumption per kilogram (VO_2_/kg), maximum heart rate (HR_max_), oxygen pulse (VO_2_/HR), minute ventilation relative to oxygen consumption (VE/VO_2_), and minute ventilation relative to carbon dioxide production (VE/VCO_2_) for each subject.

All assessments were conducted by the same trained therapist. The order of the four tests—TMS, 6MWT, PFT, and CPET—was randomized using a random number table to counterbalance potential order effects. All testing sessions were scheduled between 3:00 PM and 6:00 PM. A washout period of at least 48 h was implemented between consecutive tests. Participants were instructed to fast for at least 1 h prior to each test and to abstain from consuming caffeinated or alcoholic beverages.

### 2.3 Diaphragmatic MEP Testing Protocol

**FIGURE 1:**
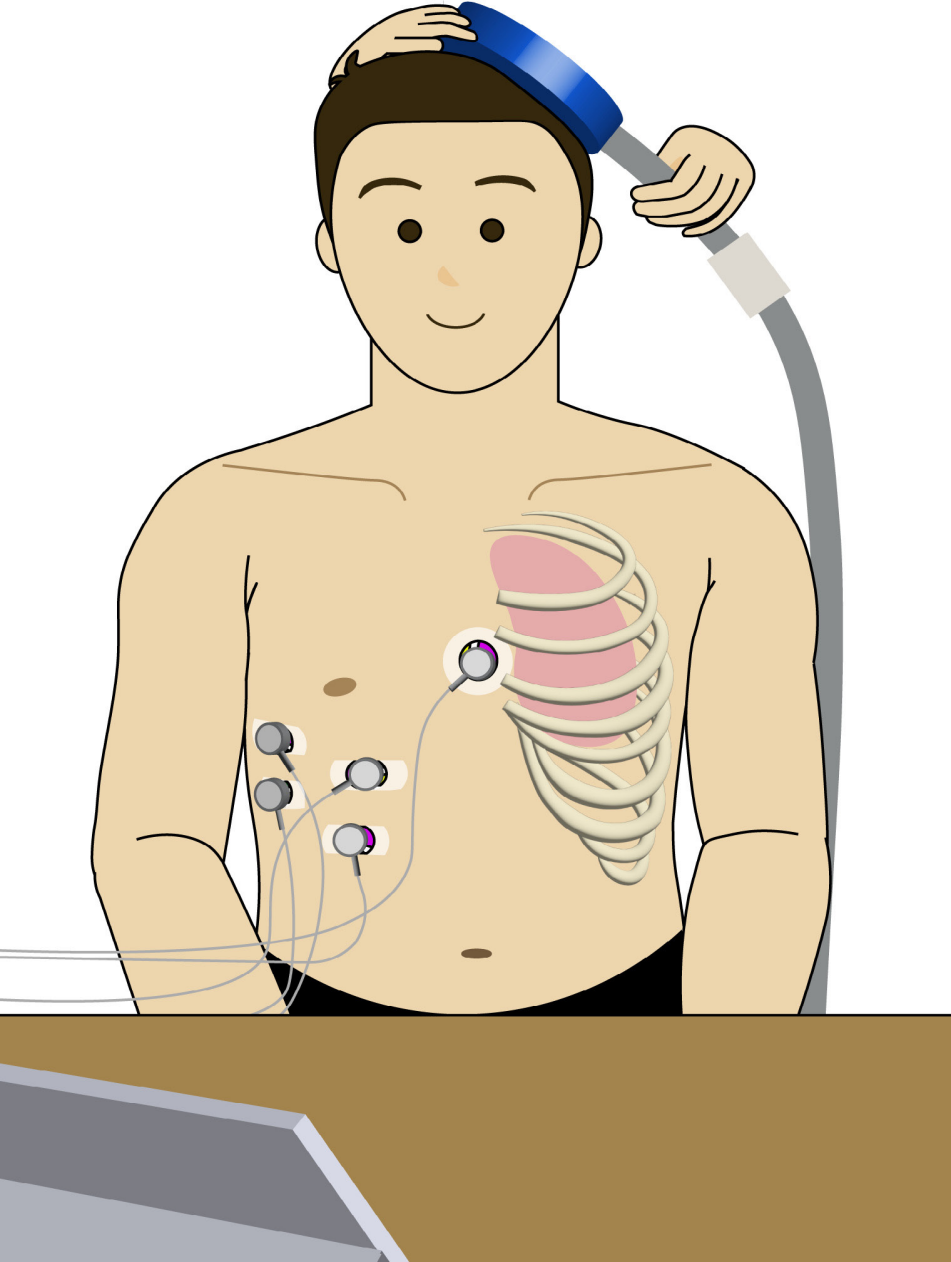
Schematic diagram of the experiment. Participant was seated comfortably with eyes open, maintaining relaxed breathing. A circular TMS coil (70 mm diameter) was positioned tangentially to the scalp at a 45° angle to the midline over the left hemisphere, targeting the diaphragmatic motor cortical representation (approximately 4 cm lateral and 1 cm anterior to Cz). Surface EMG electrodes were placed on the right chest wall (contralateral to stimulation) between the sixth and eight intercostal spaces along the anterior axillary line. A biofeedback system displayed real‐time respiratory waveforms to ensure stimulation was delivered at end‐exhalation. TMS pulses were single‐pulse, biphasic waveforms. Stimulation intensity during data acquisition was set at 120% of the individually determined resting motor threshold (RMT).

The participant was seated comfortably in a chair with back support, maintaining a relaxed posture with hands resting on their thighs. Natural breathing cycles were displayed through a biofeedback software (Beijing SensEcho Technology Co. Ltd., Beijing, China), with the inspiratory and expiratory phases presented as waveforms on a screen. Throughout the TMS session, participants were continuously monitored for any adverse effects, such as headache, neck discomfort, or scalp sensations. A schematic diagram of the experimental setup is presented in Figure 1.

#### 2.3.1 Cortical Localization

Scalp localization was performed using the international 10‐20 system, with the Cz point identified and marked at the vertex. A systematic search grid was established with 1‐cm intervals, centered on the region 4 cm lateral and 3 cm anterior to Cz. According to established literature [4, 5, 14], the optimal stimulation site for the diaphragmatic motor cortex is typically located within the approximate region of 4 cm lateral and 1 cm anterior to Cz. Potential stimulation points within this defined grid were marked for subsequent evaluation.

#### 2.3.2 Electrode Placement and Electromyography (EMG)

Two pairs of silver‐chloride electrodes with 5‐mm‐diameter recording surfaces (Model X‐1, Hangzhou Xunda Radio Appliances Co. Ltd., Hangzhou, China) were positioned on the right chest wall in a bipolar arrangement between the sixth and eighth intercostal spaces along the anterior axillary line. The ground electrode was placed on the xiphoid process, with an interelectrode distance of 3 cm. This recording configuration has been previously validated for diaphragm EMG measurements during TMS and has been demonstrated to avoid major sources of signal contamination [5, 14, 15]. The raw EMG signals were amplified with a band‐pass filter setting of 3 Hz to 3 kHz, digitized at a sampling rate of 2048 Hz, and processed with a 50‐Hz notch filter. All data were stored on disk for subsequent offline analysis.

#### 2.3.3 Determining the Hotspot

Using a circular coil, the area on the left side of the cortex was stimulated sequentially at 80% of the maximal magnetic output (MSO). The coil was applied tangentially to the scalp of the patient. Each stimulation was performed at the end of exhalation, with at least five complete breathing cycles between two consecutive stimulations. When at least one of the two surface electrode channels showed a clear MEP with maximum amplitude, this cortical area was marked as the hotspot. If 80% of the MSO failed to induce an MEP, then the stimulation intensity was increased by 5% of the MSO each time in the targeted area until it reached 100% of the MSO.

#### 2.3.4 Diaphragmatic Resting Motor Threshold (DRMT)

After locating the diaphragm's corresponding cortical hotspot in a subject, the intensity was reduced by 5% of the MSO each time until MEPs appeared in three out of five consecutive stimulations. This stimulation intensity was identified as the resting motor threshold (RMT) for the subject's diaphragm.

#### 2.3.5 Measurement of Cortical‐Diaphragm Pathway Parameters

Following the determination of diaphragmatic resting motor threshold (DRMT), 15 single‐pulse TMS stimuli were delivered at 120% RMT intensity over the identified hotspot. If the calculated stimulation intensity exceeds the MSO, stimulation will be performed at 100% MSO intensity. To ensure signal stability and minimize carryover effects, consecutive stimuli were separated by a minimum of three complete respiratory cycles (approximately 25–35 s). %MSO was used for hotspot localization to provide a standardized absolute intensity across participants, whereas %RMT was used for data acquisition to normalize stimulation intensity to individual cortical excitability, reducing interindividual variability and enabling comparable assessment of evoked responses. All participants reported good tolerance to the procedure, with no serious TMS‐related adverse events recorded.

#### 2.3.6 MEP Data Analysis

**FIGURE 2:**
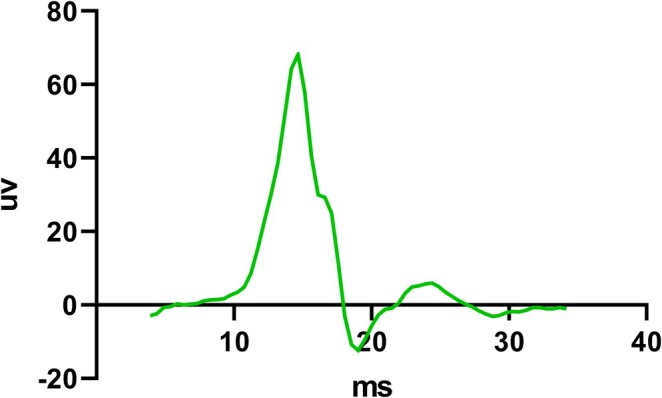
An example motor evoked potential (MEP) of the diaphragm induced by circular coil. The trace shows an example of the mean MEPs of the diaphragm averaged over 15 stimulation frames after transcranial magnetic stimulation (TMS). MEP latency (ms) was measured from the stimulus artifact to the onset of the response; MEP amplitude (μV) was measured as the peak‐to‐peak voltage difference. Stimulation parameters: single‐pulse, biphasic waveform; intensity: 120% RMT; delivered at end‐exhalation.

MEPs were analyzed offline using a custom MATLAB script (MATLAB R2013b, The MathWorks, Natick, MA, USA). The EMG data were demeaned, and the mean peak‐to‐peak amplitude (μV) and mean latency (ms) were calculated from the 15 MEPs obtained at 120% RMT intensity. For each participant, the channel demonstrating the larger mean amplitude was selected for final analysis to determine the cortical motor evoked potential amplitude (CMEPA) and the cortical motor evoked potential latency (CMEPL) of the diaphragm. An example motor evoked potential (MEP) of the diaphragm induced by circular coil is illustrated in Figure 2.

## 3 Data Analysis and Statistics

Statistical analysis was performed using SPSS 22.0 software (PASW Statistics for Windows, Version 22.0. Chicago: SPSS Inc.). Continuous variables were initially assessed for normality using the Shapiro–Wilk test. Normally distributed data are presented as mean ± standard deviation (SD), whereas non‐normally distributed data are expressed as median with interquartile range (IQR = 25th–75th percentiles).

Pearson correlation (for normally distributed data) or Spearman correlation (for non‐normally distributed data) was used to examine the associations between DRMT, CMEPA, and CMEPL with parameters from 6MWT, PFT, and CPET. Given the large number of correlation tests performed (*n* = 54), the Benjamini–Hochberg false discovery rate procedure was applied to correct for multiple comparisons, with a *q*‐value < 0.05 defined as statistically significant. Subsequently, to identify independent predictors of DRMT and CMEPL, stepwise regression analysis was conducted using parameters that remained significant after the aforementioned correction. The criteria for variable inclusion and exclusion were set at *p* < 0.05 and *p* > 0.10, respectively. Correlation coefficients (*r*) were used to describe the strength of linear association, with conventional thresholds applied: |*r*| > 0.75 indicated a strong association, 0.5 ≤ |*r*| ≤ 0.75 a moderate association, and |*r*| < 0.5 a weak association [15, 16].

## 4 Results

All 17 subjects completed all the experiments. All participants completed the 6MWT without a rest break. In the CPET, 10 of the 17 participants met the predetermined heart rate criterion for test termination, whereas 7 discontinued due to reaching maximal fatigue levels. The median DRMT, CMEPL, and CMEPA were 75 (67.5, 82.5), 13.67 (11.7, 15.6), and 111.3 (82.7, 155.8), respectively. Basic information of the subjects, along with key parameters from the TMS test, 6MWT, PFT, and CPET, is presented in Table 1.

**TABLE 1:** Basic information of the subjects and tests.

Age (years)	22.4 ± 0.8
Gender (*n*)	Male 8 Female 9
BMI (kg/m^2^)	21.4 ± 0.7
DRMT median (IQR)	75 (67.5, 82.5)
CMEPA (μV) median (IQR)	111.3 (82.71, 55.8)
CMEPL (ms) median (IQR)	13.67 (11.7, 15.6)
6MWT	
Distance (m)	612 ± 10.8
Distance_pred_%	79.8 ± 1.1
6MWT‐RR_change_ median (IQR)	14 (7.5, 18)
6MWT‐HR_change_ median (IQR)	51 (41, 69)
PFT	
FEV1(L) median (IQR)	3.18 (2.8, 4.1)
FEV1_pred_%	91.7 ± 2.1
FVC(L) median (IQR)	3.5 (3.2, 4.5)
FVC_pred_%	89 ± 1.4
FEV1/FVC_pred_%	103.1 ± 1.5
PEF(L/S) median (IQR)	5.8 (5.1, 7.6)
CPET	
WR_max_ (W) median (IQR)	119 (95 171)
VO_2max_ (L/min) median (IQR)	1.3 (1.0, 1.7)
VO_2_/kg (mL/min/kg) median (IQR)	25 (18.5, 27.5)
HR_max_ median (IQR)	160 (152.5, 167.5)
VO_2_/HR median (IQR)	8.0 (6.9, 10.3)
VE/VO_2_ median (IQR)	34.4 (30.5, 36.9)
VE/VCO_2_ median (IQR)	26.8 (24.6, 28.5)

Abbreviations: 6MWT, 6‐min walk test; 6MWT‐HR_change_, changes in heart rate during 6‐min walk test; 6MWT‐RR_change_, changes in respiratory rate during 6‐min walk test; CMEPA, cortical motor evoked potential amplitude; CMEPL, cortical motor evoked potential latency; CPET, cardiopulmonary exercise test; DRMT, diaphragmatic resting motor threshold; FEV1, forced expiratory volume in the first second; FEV1/FVC_pred%_, forced expiratory volume in 1 s to forced vital capacity ratio, expressed as a percentage of the predicted value; FEV1_pred_%, forced expiratory volume in 1 s as a percentage of the predicted value; FVC, forced vital capacity; FVC_pred_%, forced vital capacity as a percentage of the predicted value; HR_max_, maximum heart rate; PEF, peak expiratory flow; PFT, pulmonary function test; VE/VCO_2_, minute ventilation relative to carbon dioxide production; VE/VO_2_, minute ventilation relative to oxygen consumption; VO_2_/HR, oxygen pulse; VO_2_/kg, oxygen consumption per kilogram; VO_2max_, maximum oxygen uptake; WR_max_, maximum work rate.

### 4.1 Correlation Analysis

Correlation analyses were conducted between DRMT, CMEPA, and CMEPL and various parameters from the 6MWT, PFT, and CPET. The strongest observed association was between CMEPL and HR_max_, which showed a strong positive correlation (*r* = 0.750, *p* = 0.001). CMEPL also demonstrated moderate positive correlations with VO_2max_ (*r* = 0.589, *p* = 0.013), FEV1/FVC_pred_% (*r* = 0.485, *p* = 0.049), and VO_2_/HR (*r* = 0.497, *p* = 0.043).

For DRMT, moderate positive correlations were observed with FEV1 (*r* = 0.590, *p* = 0.013), FVC (*r* = 0.591, *p* = 0.013), and VO_2max_ (*r* = 0.501, *p* = 0.041), as well as a moderate negative correlation with the change in respiratory rate during the 6MWT (*r* = −0.513, *p* = 0.035). A weak positive correlation was also noted between DRMT and FVC%pred (*r* = 0.483, *p* = 0.050).

**FIGURE 3:**
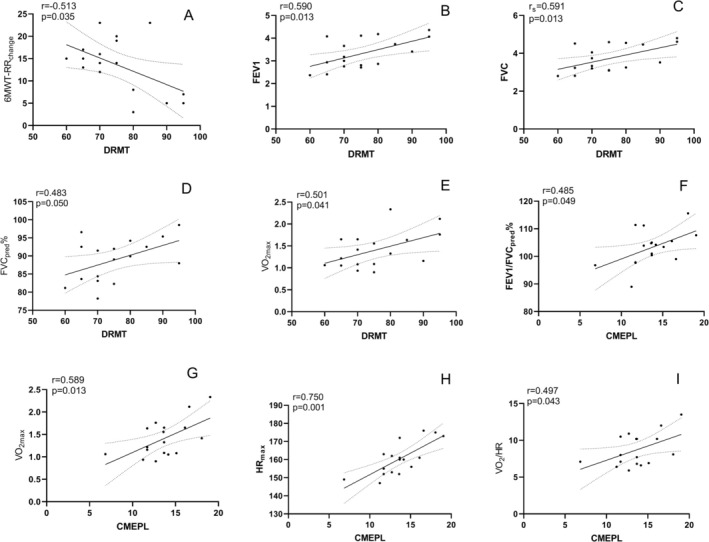
The statistically significant correlation results between DRMT, CMEPA, and CMEPL and various parameters from the 6MWT, PFT, and CPET Scatter plots showing the significant associations with solid lines representing regression lines and dashed lines indicating 95% confidence intervals. (A) DRMT and 6MWT‐RRchange, (B) DRMT and FEV1, (C) DRMT and FVC, (D) DRMT and FVC_pred_%, (E) DRMT and VO_2max_, (F) CMEPL and FEV1/FVC_pred_%, (G) CMEPL and VO_2max_, (H) CMEPL and HR_max_, (I) CMEPL and VO_2_/HR. 6MWT‐RR_change_, changes in respiratory rate during 6‐min walk test; CMEPL, cortical motor evoked potential latency; DRMT, diaphragmatic resting motor threshold; FEV1/FVC_pred_%, forced expiratory volume in 1 s to forced vital capacity ratio, expressed as a percentage of the predicted value; FEV1, forced expiratory volume in the first second; FVC, forced vital capacity; FVC_pred_%, forced vital capacity as a percentage of the predicted value; HR_max_, maximum heart rate; VO_2_/HR, oxygen pulse; VO_2max_, maximum oxygen uptake.

The statistically significant correlation results are presented in Figure 3. The complete correlation matrices between all TMS parameters (DRMT, CMEPA, and CMEPL) and all cardiopulmonary function metrics from the 6MWT, PFT, and CPET are provided in Figures S1–S3, respectively.

### 4.2 Stepwise Regression Analysis

To identify independent predictors of DRMT and CMEPL and to build parsimonious models from a dataset containing multiple highly correlated cardiopulmonary parameters, we performed stepwise regression analysis. Parameters showing significant correlations (*p* < 0.05) with the respective dependent variables in the aforementioned correlation analyses were included as candidate independent variables. The analysis revealed that FEV1 was an independent predictor of DRMT (B = 9.442, *p* = 0.013; Table 2). Furthermore, HR_max_ was identified as an independent predictor of CMEPL (B = 0.237, *p* = 0.001; Table 2).

**TABLE 2:** Stepwise regression analyses for DRMT and CMEPL (*n* = 17).

Dependent variable	Predictor	B (SE)	*t*	*p*	Model fit
DRMT	Constant	44.184 (11.309)	3.907	0.001	*R* ^2^ = 0.348, adj. *R* ^2^ = 0.304
	FEV1	9.422 (3.332)	2.828	0.013	F_1,15_ = 7.998, *p* = 0.013
CMEPL	Constant	−24.328 (8.659)	−2.809	0.013	*R* ^2^ = 0.563, adj. *R* ^2^ = 0.534
	HR_max_	0.237 (0.054)	4.394	0.001	F_1,15_ = 19.304, *p* = 0.001

Abbreviations: Adj.*R* ^2^, adjusted R‐squared; CMEPL, cortical motor evoked potential latency; DRMT, diaphragmatic resting motor threshold; FEV1, forced expiratory volume in 1 s; HR_max_, maximal heart rate.

## 5 Discussion

This study demonstrates that standard measures of cardiopulmonary function are associated with distinct parameters of corticospinal diaphragm pathway excitability and conduction in healthy adults. Among these measures, FEV1 and HR_max_ emerged as the two strongest independent predictors, highlighting potential links between peripheral cardiopulmonary status and central respiratory motor pathways. Overall, better cardiopulmonary function was associated with higher DRMT and longer corticospinal conduction latency.

### 5.1 Association Between FEV1 and DRMT

FEV1 was independently and positively associated with DRMT (B = 9.442, *p* = 0.013; Table 2; see also Figure 3B for the correlation), indicating that individuals with higher pulmonary ventilation capacity tended to exhibit higher motor thresholds, a pattern consistent with lower cortical excitability. This association suggests a possible relationship between peripheral respiratory efficiency and central respiratory motor responsiveness. To contextualize this pattern, studies related to COPD have reported the opposite association: Lower FEV1 is often accompanied by a reduced DRMT [17, 18], which has been interpreted as a compensatory increase in cortical excitability in response to chronic respiratory loading. These observations from disease states offer a useful contrast but do not imply mechanistic equivalence. Instead, they highlight that the “high FEV1 → high DRMT” pattern observed in healthy adults may reflect a non‐pathological coupling between pulmonary function and cortical responsiveness [19, 20]. A plausible hypothesis is that when respiratory mechanics are efficient, the central nervous system may not require heightened excitability to support routine ventilatory demands [21, 22]. Acute intermittent hypercapnic‐hypoxia (AIHH) enhances corticospinal diaphragm neural transmission, whereas hypoxia alone has no such effect, suggesting that CO_2_ levels and chemosensory input play a role in regulating the excitability of the corticospinal diaphragm pathway [23]. Based on this, it is speculated that a higher FEV1 may be associated with better ventilatory efficiency and more stable blood gas homeostasis, which in turn may be linked to lower cortical excitability. This interpretation remains speculative and should be tested in future mechanistic studies. Importantly, because FEV1 is widely available in clinical settings, its association with DRMT may offer a practical indicator for exploring central–peripheral relationships in respiratory control.

### 5.2 Association Between HR_max_ and CMEPL

HR_max_ was independently and positively associated with CMEPL (B = 0.237, *p* = 0.001; Table 2; see also Figure 3H for the correlation), with a strong positive correlation between the two (*r* = 0.750; Figure 3). Conventionally, shortened neural conduction latency is considered a marker of improved function. In contrast, our study found that healthy individuals with higher HR_max_ exhibited longer CMEPL. The present associative pattern suggests that individuals with superior exercise capacity may exhibit different conduction characteristics, potentially reflecting long‐term adaptations related to cardiovascular fitness. One hypothesis is that higher cardiopulmonary reserve is frequently accompanied by enhanced vagal tone [24], which could relate to conduction timing through brainstem pathways [25, 26]. Another possibility is that improved cerebral oxygenation in fitter individuals may support neural integration strategies that do not prioritize rapid conduction [27, 28]. These explanations are speculative and should be interpreted as hypotheses rather than mechanistic conclusions. Research about COPD studies provides a contrasting context: Chronic hypoxia in COPD has been associated with shorter CMEPL and increased cortical excitability [29, 30, 31]. These findings underscore that the pattern observed in healthy adults likely reflects non‐pathological physiological variation, rather than a compensatory mechanism. Identifying HR_max_—a core CPET parameter—as a predictor of cortical‐diaphragmatic conduction latency offers a novel associative perspective on how cardiovascular fitness may relate to corticospinal respiratory pathways.

### 5.3 Plausible Explanations for the Lack of Correlation With CMEPA

The lack of correlation between CMEPA and all measured parameters may be attributed to interference from neural oscillatory synchrony: Gamma‐band (30–50 Hz) oscillations in the motor cortex significantly influence MEP amplitude [32, 33]. Furthermore, technical factors such as intercostal muscle co‐activation and electrode placement variability also contributed to the variability [4, 14, 34].

## 6 Limitation

First, our study represents a pilot investigation with a limited sample size comprising only young, healthy individuals. These findings are preliminary and may not be generalizable to older adults or patients with cardiopulmonary conditions. Future research should expand the sample size and include diverse age groups as well as populations with cardiopulmonary diseases.

Second, due to experimental constraints, this study did not record EMG signals from the diaphragm ipsilateral to the TMS stimulation. In a study of 30 healthy male volunteers [14], TMS applied to either cerebral hemisphere elicited MEPs with significantly larger amplitude and shorter latency in the contralateral versus ipsilateral diaphragm. Therefore, our study prioritized recording MEPs from the diaphragm contralateral to the stimulation site.

Third, the study did not document participants' exercise levels or habitual physical activity, factors that could influence both cardiopulmonary function and cortical excitability. However, it is understood that none of the participants were professional athletes or engaged in regular, structured exercise routines.

Finally, the results are presented using univariate regression models, which may represent a simplification of more complex physiological relationships. In preliminary analyses, we did attempt to construct multivariate models incorporating other potentially important variables such as FVC and VO_2_/HR. However, given the limited sample size (*n* = 17) and the high degree of physiological collinearity among these cardiopulmonary parameters, the multivariate models became unstable and difficult to interpret. Consequently, we adopted a conservative strategy, reporting the most robust univariate associations to clearly elucidate the core roles of FEV1 and HR_max_ as independent predictors. Future studies with larger sample sizes could employ more flexible statistical approaches, such as ridge regression or structural equation modeling, to integrate these highly correlated variables and more comprehensively unravel the complex interrelationships.

## 7 Conclusions

This study represents the first comprehensive investigation into the relationship between the corticospinal diaphragm pathway and cardiopulmonary function in healthy individuals. Our findings demonstrate significant correlations between this pathway and cardiopulmonary metrics, particularly involving FEV1 and HR_max_. Specifically, healthier individuals with better pulmonary function (higher FEV1) exhibited lower cortical excitability, reflected in a higher DRMT, whereas those with greater cardiopulmonary reserve (higher HR_max_) showed prolonged CMEPL. These results indicate that in healthy individuals, superior cardiopulmonary function is associated with a higher DRMT and a longer CMEPL. This pattern of association is consistent with the possibility of coordinated associations between cardiopulmonary and neural systems. Future studies are needed to explore the underlying mechanisms and to evaluate whether these associations hold diagnostic or therapeutic relevance.

## Author Contributions

**Long‐ping Wang:** writing – review and editing, writing – original draft, visualization, software, methodology, investigation, formal analysis, data curation. **Jing Chen:** methodology, visualization, investigation. **Cheng Wu:** methodology, investigation. **Shan‐tong Yao:** methodology, investigation. **Jin‐ze Tan:** methodology, investigation. **Shuang Guo:** methodology, investigation. **Qian Ding:** writing – review and editing, methodology, investigation, formal analysis, funding acquisition, data curation. **Guang‐qing Xu:** writing – review and editing, resources, project administration, funding acquisition, conceptualization.

## Funding

This study was supported by the National Natural Science Foundation of China (82272588 [to Guang‐qing Xu], 82572917 [to Guang‐qing Xu], and 82102678 [to Qian Ding]) and Guangdong Basic and Applied Basic Research Foundation (2026A1515012150 [to Qian Ding]), and Guangdong Medical Research Foundation (A2024500 [to Qian Ding] and B2026188 [to Qian Ding]).

## Ethics Statement

The study was approved by the Ethics Committee of Guangdong Provincial People's Hospital (Ethical number: KY2024‐1370‐01), and all subjects provided informed consent.

## Conflicts of Interest

The authors declare no conflicts of interest.

## Supporting information


**Figure S1:** Supporting Information.

**Figure S2:** Supporting Information.

**Figure S3:** Supporting Information.

## Data Availability

The data that support the findings of this study are available from the corresponding author upon reasonable request.
