# Supplementary figures and images for: The Relationship Between the Cortico‐Diaphragmatic Conduction Pathway and Cardiopulmonary Function in Healthy Individuals
Source: Clin Respir J. 2026 May 24;20(5):e70198. doi: 10.1111/crj.70198 (PMC13238803; doi:10.1111/crj.70198)

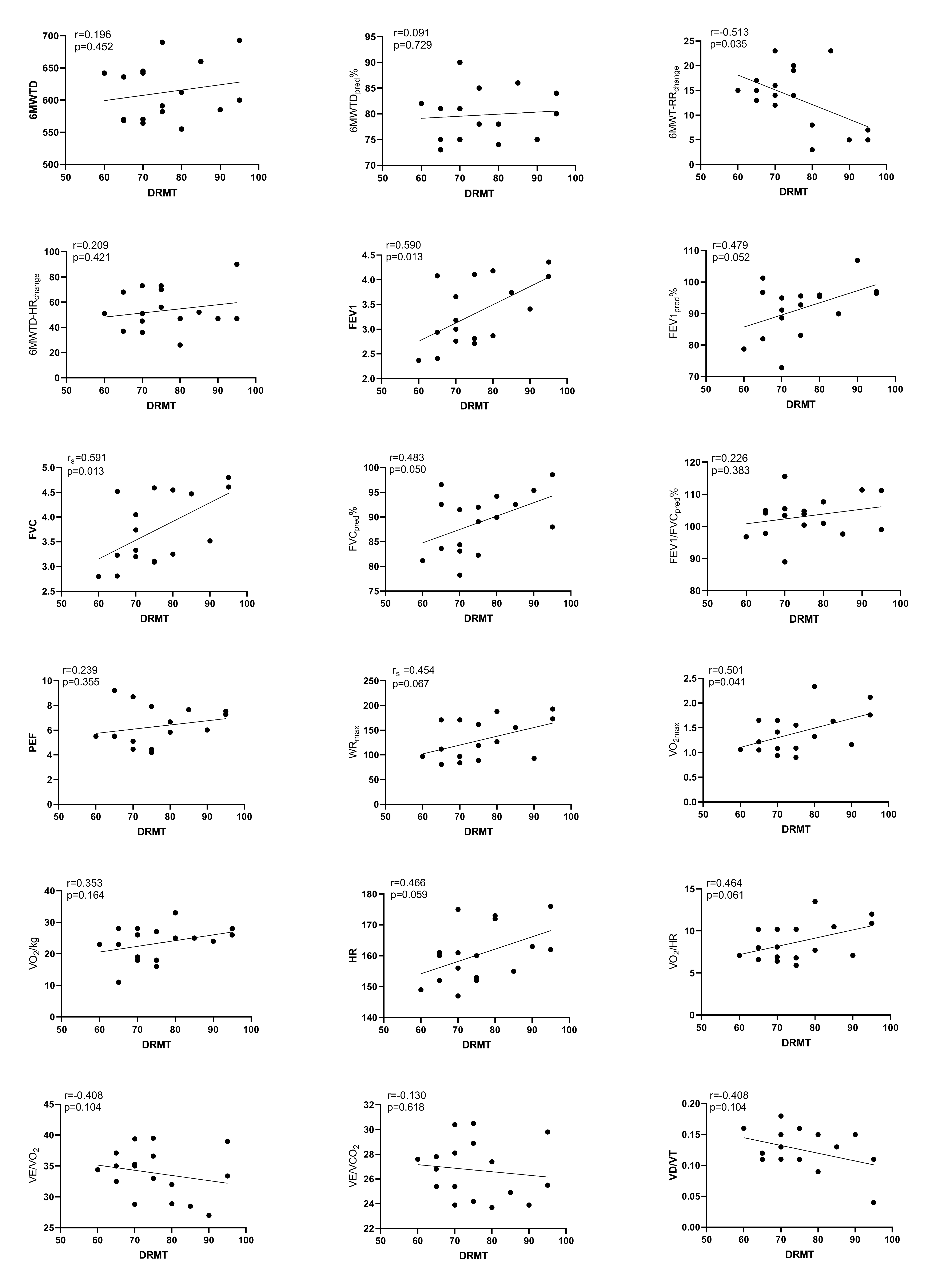

Supplement: Supplementary file 1 — Figure S1: Supporting Information. [file CRJ-20-e70198-s003.tif]

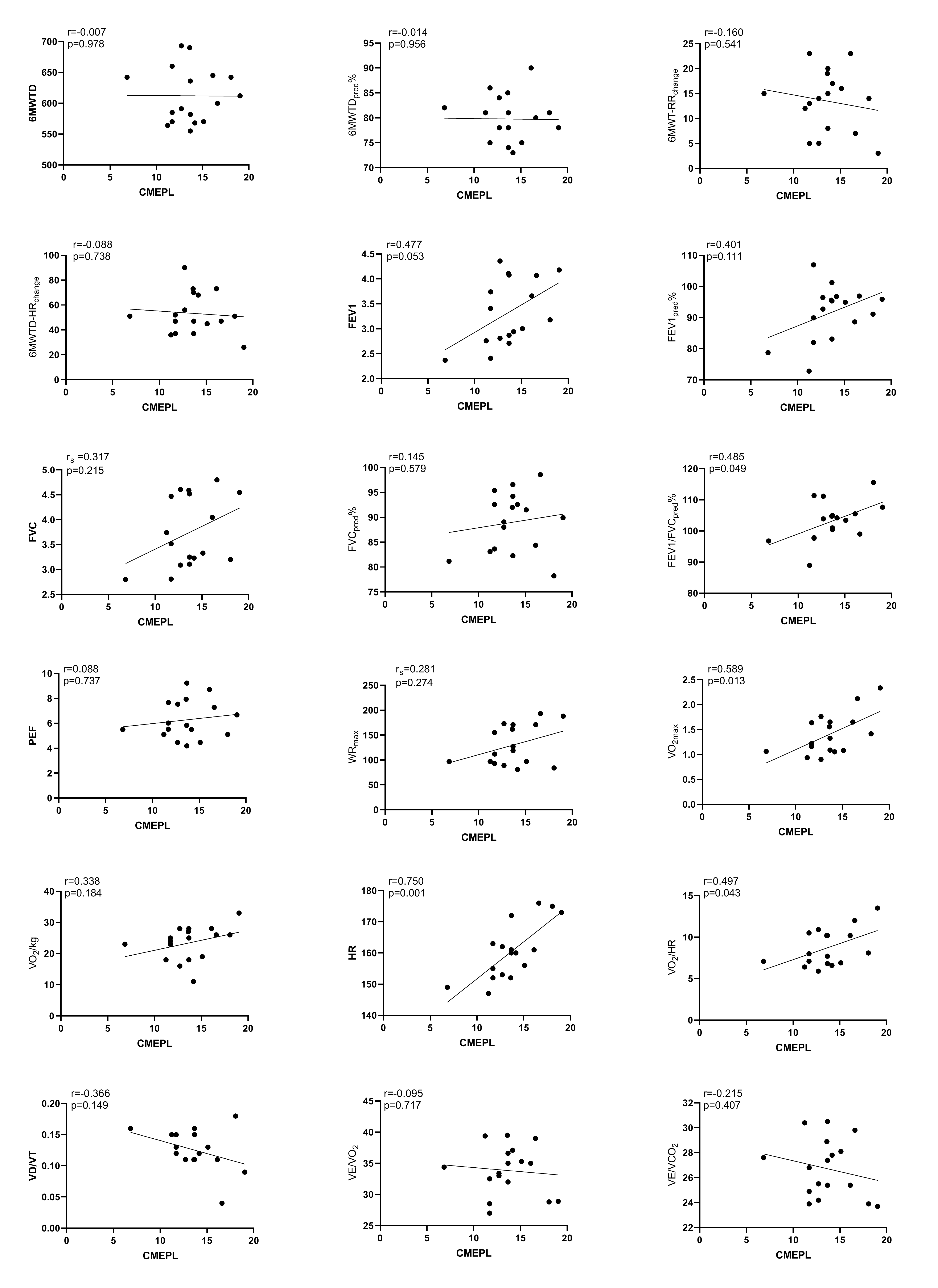

Supplement: Supplementary file 2 — Figure S2: Supporting Information. [file CRJ-20-e70198-s002.tif]

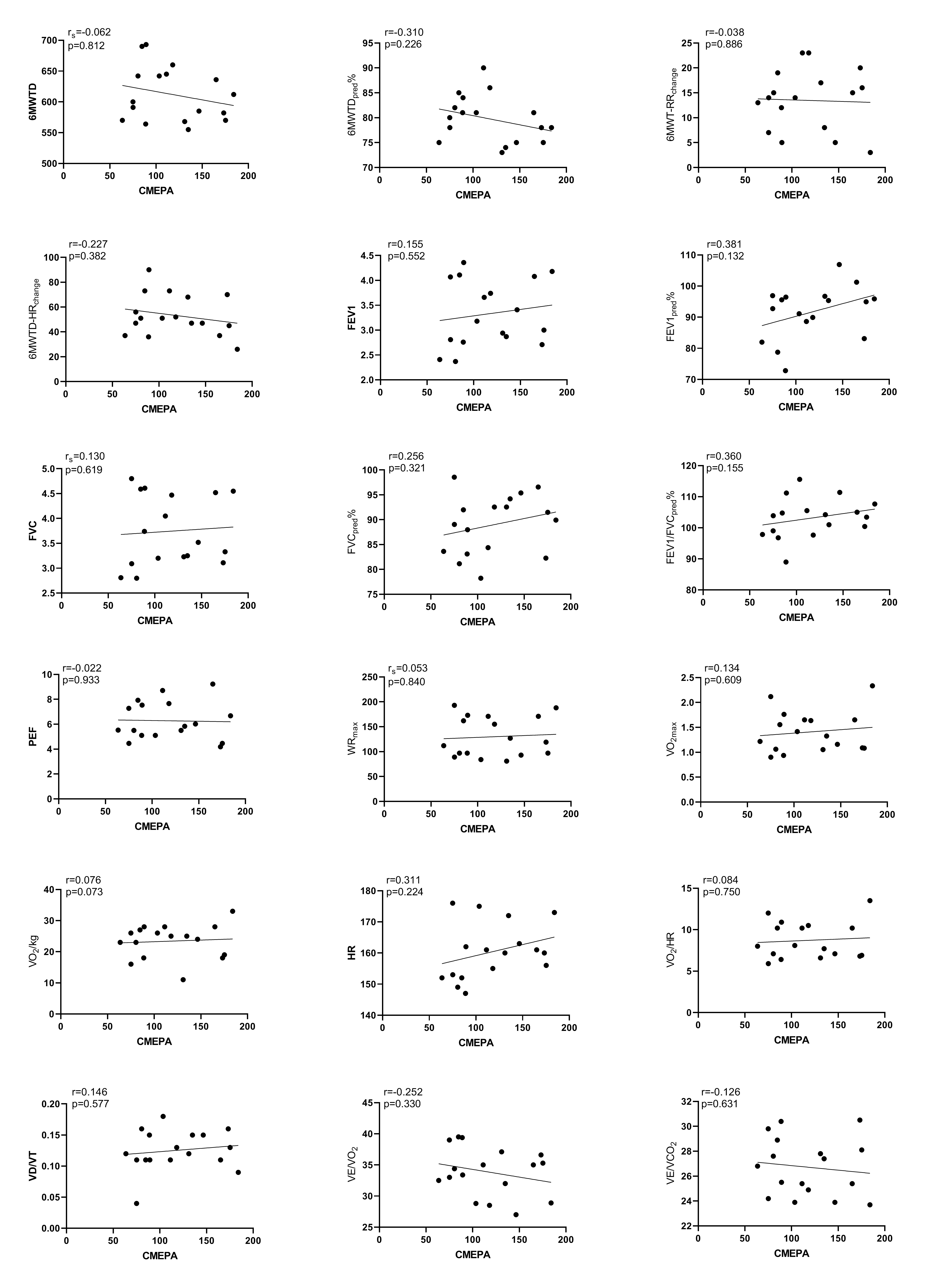

Supplement: Supplementary file 3 — Figure S3: Supporting Information. [file CRJ-20-e70198-s001.tif]
